# Myelin oligodendrocyte glycoprotein antibody titers by fixed cell-based assay: positive predictive value and impact of sample collection timing

**DOI:** 10.3389/fneur.2024.1380541

**Published:** 2024-03-14

**Authors:** Adrian Budhram, Dalia L. Rotstein, Liju Yang, E. Ann Yeh

**Affiliations:** ^1^London Health Sciences Centre, Department of Clinical Neurological Sciences, Western University, London, ON, Canada; ^2^London Health Sciences Centre, Department of Pathology and Laboratory Medicine, Western University, London, ON, Canada; ^3^Division of Neurology, Department of Medicine, University of Toronto, Toronto, ON, Canada; ^4^Department of Paediatrics (Neurology), Hospital for Sick Children, Division of Neuroscience and Mental Health, The Hospital for Sick Children Research Institute, University of Toronto, Toronto, ON, Canada

**Keywords:** autoantibody, MOG, MOGAD, demyelinating disease, autoimmune neurology

## Abstract

**Introduction:**

In January 2023, our laboratory began performing serum myelin oligodendrocyte glycoprotein antibody (anti-MOG) titers by fixed cell-based assay (CBA). As a quality assurance (QA) assessment, we evaluated titer positive predictive value (PPV) as well as impact of sample collection timing on titers.

**Methods:**

Among patients who underwent antibody titers to distinguish between low-positive (<1:100) and clear-positive (≥1:100) anti-MOG, records were reviewed to classify results as true-positive (TP) or false-positive (FP) and facilitate PPV calculation. Timing of sample collection relative to administration of immunotherapy and symptom onset was determined for TP results.

**Results:**

Overall PPV of anti-MOG was 70/85 (82%). The PPV of low-positive anti-MOG was significantly lower than clear-positive anti-MOG (72% vs. 95%, *p* = 0.009). The difference in PPV between low-positive and clear-positive anti-MOG was significant among adults tested, but not children. Among patients with TP anti-MOG, the proportion who received immunotherapy prior to sample collection was significantly higher and median time from symptom onset to sample collection was significantly longer for low-positive compared to clear-positive results.

**Conclusion:**

Overall PPV of anti-MOG testing by fixed CBA was reasonably high. The PPV of low-positive anti-MOG was significantly lower than clear-positive anti-MOG. This was driven by the significantly lower PPV of low-positive anti-MOG in adults, possibly reflecting the lower prevalence of MOG antibody-associated disease among adults tested. Timing of sample collection relative to administration of immunotherapy and symptom onset may substantially impact titers, indicating that testing should ideally be performed prior to immunotherapy and close to time of attack.

## Introduction

Myelin oligodendrocyte glycoprotein antibody (anti-MOG) is the defining biomarker of MOG antibody-associated disease (MOGAD), an inflammatory demyelinating disease that is distinct from multiple sclerosis (MS). Core clinical demyelinating events that have been identified in MOGAD are optic neuritis, myelitis, inflammatory brainstem/cerebellar syndromes, and inflammatory cerebral syndromes that include acute disseminated encephalomyelitis (ADEM) and cerebral cortical encephalitis (CCE) (1–5). Testing for anti-MOG by cell-based assay (CBA), which can be performed using live or fixed cells, has been reported to have high overall specificity for MOGAD and is the recommended methodology to test for this antibody in clinical laboratories (5–7). However, studies using live CBA have highlighted that low serum titers of anti-MOG have lower positive predictive value (PPV) for MOGAD, and may be found in patients with alternative diagnoses such as MS or stroke (8–10).

Consequently, the recently proposed 2023 MOGAD criteria recommend that serum anti-MOG titers be included in results reported out by clinical laboratories, and outline additional supportive criteria for making a diagnosis of MOGAD in a patient with only a “low-positive” antibody result (5). For fixed CBA, these criteria define “low-positive” as an anti-MOG titer that is at least 1:10 and less than 1:100, and “clear-positive” as an anti-MOG titer that is equal to or greater than 1:100 (5). Prior to development of these criteria the London Health Sciences Centre (LHSC) Clinical Immunology laboratory, which is a hospital-affiliated laboratory in Canada that provides neural antibody testing to clinicians in Southwestern Ontario as well as other academic and community practice settings across the province, performed serum testing for anti-MOG by fixed CBA but did not offer antibody titers. However, to align with the recent MOGAD criteria we began performing serum anti-MOG titers by fixed CBA in January 2023.

Following this operational change, assurance that anti-MOG titers by fixed CBA represent an improvement in the quality of anti-MOG testing we offer is essential to confirm clinical utility of titers we are now reporting as part of routine laboratory practice, and justify their burden on laboratory cost and workflow (11). This is particularly important for our laboratory because the recommendation by the 2023 MOGAD criteria to perform anti-MOG titers is primarily based on studies of titers using live CBA, which are not identical to titers using fixed CBA (8, 9, 12, 13). With respect to how to best evaluate the clinical utility of fixed CBA anti-MOG titers, determinations of sensitivity and specificity are limited by the lack of an independent diagnostic gold standard to effectively discriminate between true-negative and false-negative anti-MOG results (5). Yet PPV, which reflects the probability that a patient with a positive test result truly has the disease of interest, is a measure of diagnostic test performance that remains feasible to determine and is of practical utility to clinicians serviced by our laboratory (14). For this reason, we evaluated the PPV of low-positive versus clear-positive fixed CBA anti-MOG results reported out from LHSC Clinical Immunology laboratory after one year, as a quality assurance (QA) assessment of this recent test offering.

## Methods

**Figure 1:**
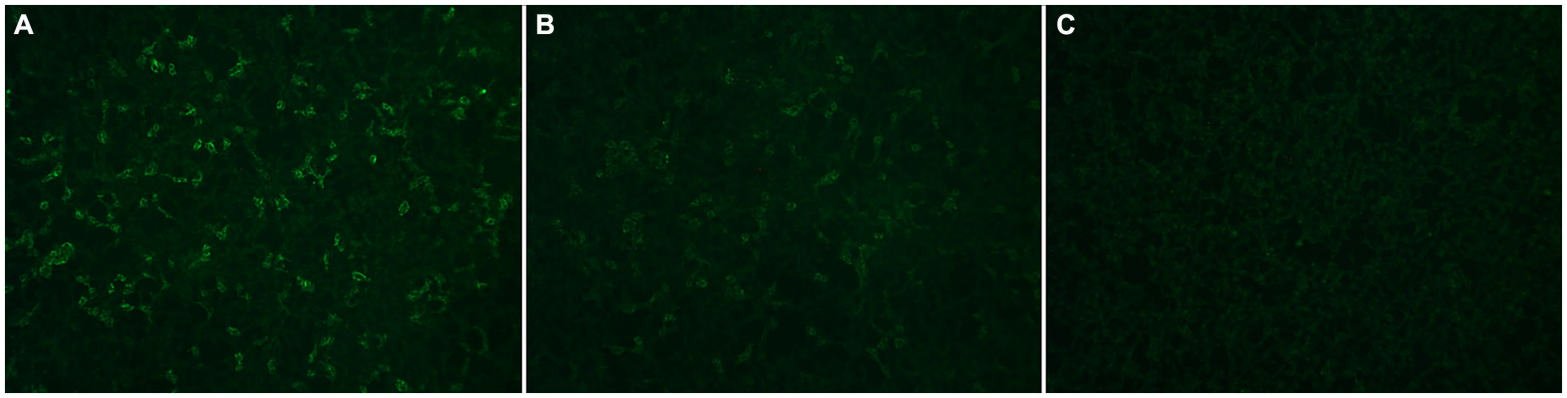
Representative images of anti-MOG results classified qualitatively as Positive **(A)**, Weak Positive **(B)**, and Negative **(C)** at London Health Sciences Centre Clinical Immunology laboratory.

We reviewed all patients with positive serum fixed CBA anti-MOG results from LHSC Clinical Immunology laboratory who underwent antibody titers between January 2023 and December 2023 as part of routine laboratory practice. For patients with multiple positive anti-MOG results over this time period only the first positive result was included in this assessment, because antibody titers were only performed on this initial positive result; this in keeping with our primary intent of offering anti-MOG titers to aid disease diagnosis rather than disease monitoring. Serum testing for anti-MOG by fixed CBA was initially performed at 1:10 dilution in accordance with manufacturer’s instructions (Euroimmun, Lubeck, Germany). For each patient, anti-MOG was classified qualitatively as Negative, Weak Positive or Positive at 1:10 dilution. “Weak Positive” refers to staining that is faint, but of sufficient intensity above the background to suggest a positive result (see Figure 1); the distinction between “Weak Positive” and “Positive” is made in an attempt to operationalize the subjectivity of indirect immunofluorescence interpretation in clinical laboratory practice (15). Patients with a Weak Positive or Positive anti-MOG result at 1:10 dilution were reflexed to undergo repeat testing at 1:100 dilution. Anti-MOG was then classified qualitatively as Negative, Weak Positive or Positive at 1:100 dilution. Only patients with Weak Positive or Positive anti-MOG results at 1:10 dilution were included in this QA assessment. Among them, those with a Negative anti-MOG result at 1:100 dilution were classified as “low-positive” while those with a Weak Positive or Positive anti-MOG result at 1:100 dilution were classified as “clear-positive,” in keeping with the 2023 MOGAD criteria (5). Electronic medical records of patients with positive anti-MOG results were retrospectively reviewed using eHealth Ontario, a secure provincial electronic health record information system. Patients with positive anti-MOG results who did not have pertinent assessment documented were excluded. Electronic medical record review was performed by A.B., a neurologist with fellowship training in Autoimmune Neurology, to classify each positive anti-MOG result as true-positive (TP) or false-positive (FP) for MOGAD and facilitate PPV determination. Classification was based on last documentation of the treating clinician. Patients with a core clinical demyelinating event compatible with MOGAD (i.e., optic neuritis, myelitis, inflammatory brainstem/cerebellar syndrome or inflammatory cerebral syndrome including ADEM, cerebral monofocal or polyfocal deficits, or CCE with consistent neuroimaging) and no more likely alternative diagnosis were classified as TP (5), while all other patients were classified as FP. Classification as TP did not require that the 2023 MOGAD criteria be met, because these criteria assume a difference in low-positive versus clear-positive anti-MOG results which was the focus of this QA assessment. Cases in which there was substantial diagnostic uncertainty that precluded confident classification as TP or FP were excluded. Following classification of positive anti-MOG results as TP or FP, the PPV (defined as the number of TP/the total number of positives) was calculated, both overall as well as for low-positive versus clear-positive antibody results. Because PPV is impacted by the disease prevalence in the tested population, stratified PPV calculations for adults and children (defined as <18 years of age) were also performed given the likely higher prevalence of MOGAD in children with suspected inflammatory demyelinating disease (16–18). Furthermore, because timing of sample collection relative to administration of immunotherapy as well as symptom onset has been reported to impact anti-MOG titers (19–21), the proportion of patients who received immunotherapy prior to sample collection (defined as immunotherapy administered one or more days prior to sample collection) as well as the time from symptom onset of most recent attack to sample collection was compared for low-positive versus clear-positive TP anti-MOG results.

Continuous and categorical variables were compared using Mann–Whitney U test and Fisher’s exact test, respectively. A *p*-value less than 0.05 was considered statistically significant. This evaluation was pursued as a QA assessment of a recently-implemented clinical-service basis laboratory test offering in a particular setting (LHSC Clinical Immunology laboratory). As per our institutional research ethics board, quality assurance/improvement assessments do not fall within the scope of institutional ethical review under Article 2.5 of the Tri-Council Policy Statement: Ethical Conduct for Research Involving Humans (TCPS 2). While not requiring REB review, ethical issues that may arise during QA assessments of laboratory testing at LHSC are still thoroughly considered within the Department of Pathology and Laboratory Medicine to minimize any potential risk of harm to participants and ensure that ethical requirements for the protection of human participants in Quality Assurance/Improvement are met (22).

## Results

**Figure 2:**
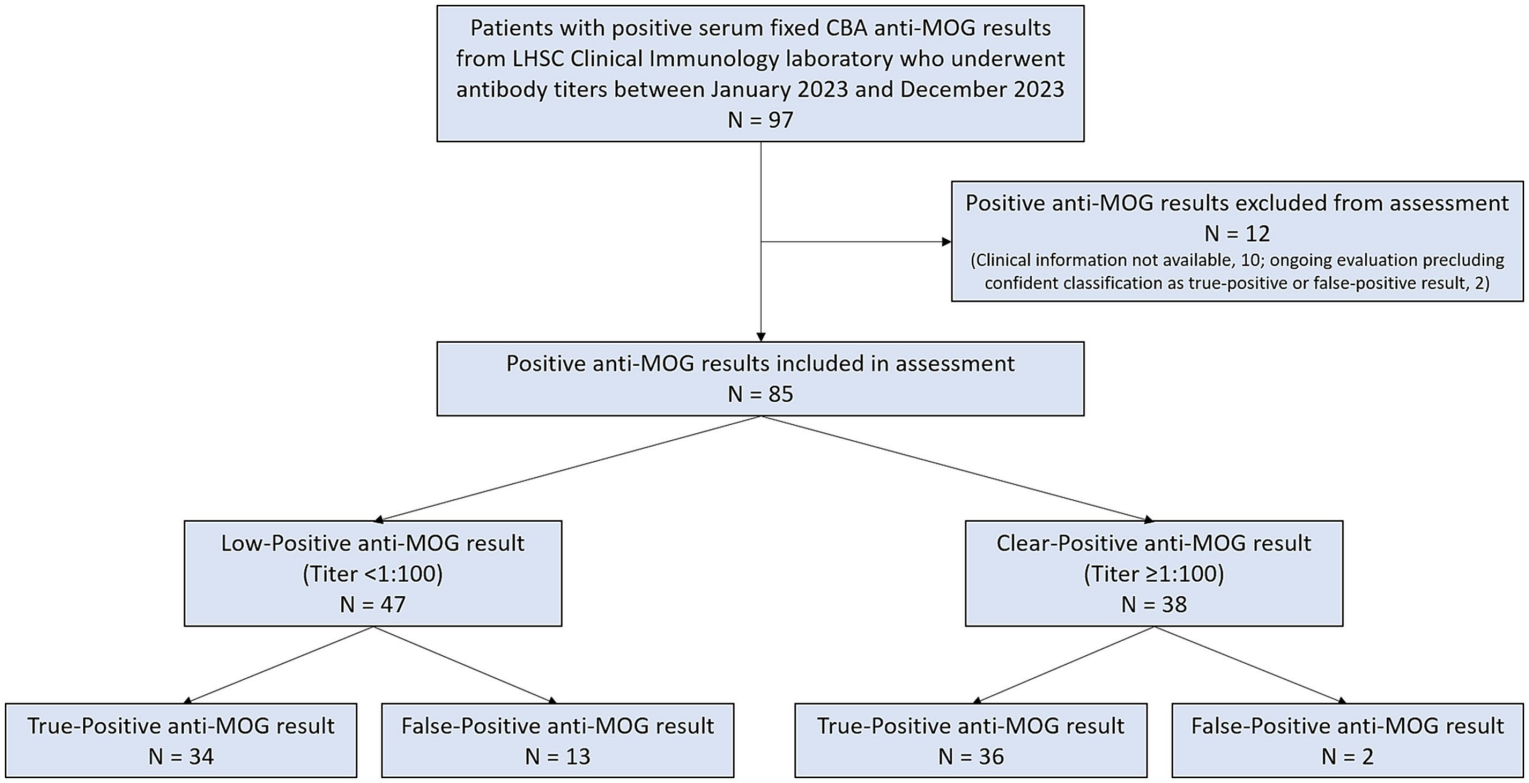
Flow diagram of positive anti-MOG results reported out from London Health Sciences Centre Clinical Immunology laboratory that were included in this assessment.

Over the 1 year period evaluated, 97 patients with positive serum fixed CBA anti-MOG results underwent antibody titers that were reported out from LHSC Clinical Immunology laboratory. Fifty-six of 97 (58%) had low-positive anti-MOG results and 41/97 (42%) had clear-positive anti-MOG results. Of 33 patients with Weak Positive anti-MOG results at 1:10 dilution, all 33 (100%) were Negative for anti-MOG at 1:100 dilution and thus classified as low-positive. Ten patients (8 with low-positive anti-MOG results, 2 with clear-positive anti-MOG results) had no assessment pertinent to their anti-MOG testing documented and were excluded from further analysis. Electronic medical records of the remaining patients were reviewed for TP or FP anti-MOG classification. Two patients (1 with a low-positive anti-MOG result, 1 with a clear-positive anti-MOG result) could not be classified as TP or FP with confidence and were also excluded from further analysis; both patients had attacks compatible with demyelination but were pending investigations to help distinguish between MOGAD and MS. This resulted in 85 patients with positive anti-MOG results who could be confidently classified as TP or FP and were thus included in this QA assessment (see Figure 2).

Demographic data of these 85 patients, both overall as well as for low-positive versus clear-positive anti-MOG results, are summarized in Table 1. Forty-seven of 85 (55%) had low-positive anti-MOG results and 38/85 (45%) had clear-positive anti-MOG results. Seventy-eight of 85 (92%) had clinical assessment documented by a neurologist specializing in MS/Neuroimmunology. Following electronic medical record review, 70 were classified as TP and 15 were classified as FP. All patients with anti-MOG results that were classified as TP were negative for anti-aquaporin-4 by fixed CBA. Of those with anti-MOG results classified as FP, 13 were low-positive (more likely alternative diagnoses identified: MS, 7; spinal cord infarction, 1; idiopathic bilateral facial palsy, 1; post-HSV anti-NMDAR encephalitis, 1; neurodevelopmental disorder with epilepsy secondary to perinatal brain injury, 1; ocular manifestations of inflammatory bowel disease, 1; systemic lupus erythematosus without neuropsychiatric involvement, 1) and 2 were clear-positive (more likely alternative diagnoses identified: neurosarcoidosis, 1; autoimmune encephalitis, 1). Patients diagnosed with MS met 2017 McDonald criteria and had typical MS lesions on MRI (8, 23, 24). No patient with a more likely alternative non-MS neuroinflammatory disease diagnosis had neuroimaging findings consistent with MOGAD (5), supporting their FP classification.

**Table 1:** Demographic data of patients with positive serum fixed CBA anti-MOG results who were included in this assessment.

	All positive anti-MOG results *N* = 85	Low-positive anti-MOG results (Titer < 1:100) *N* = 47	Clear-positive anti-MOG results (Titer ≥ 100) *N* = 38	*p*-value*
Median age at time of testing, years (range)	16.1 (0.2–77.1)	17.2 (1.1–77.1)	14.6 (0.2–77.0)	0.26
Pediatric (%)	54 (64)	28 (60)	26 (68)	0.50
Female (%)	41 (48)	21 (45)	20 (53)	0.52

*p-value refers to comparison of difference between low-positive and clear-positive anti-MOG results.

CBA, cell-based assay; MOG, myelin oligodendrocyte glycoprotein.

The PPV of anti-MOG testing is summarized in Table 2. Overall, the PPV of positive serum fixed CBA anti-MOG results reported out from our laboratory was 70/85 (82%). When stratified by titer, the PPV of low-positive anti-MOG results was significantly lower than that of clear-positive anti-MOG results (72% vs. 95%, *p* = 0.009). This significant difference appeared to be driven by the lower PPV of low-positive anti-MOG results in adults, among whom the most common alternative diagnosis identified was MS; no significant difference in the PPV of low-positive versus clear-positive anti-MOG results was observed in children. Among patients with anti-MOG results that were classified as TP, 48/70 (69%) had received immunotherapy prior to sample collection that most often consisted of corticosteroids (*N* = 48), followed by intravenous immunoglobulin (*N* = 11), rituximab (*N* = 11), plasma exchange (*N* = 5), mycophenolate mofetil (*N* = 3), and azathioprine (*N* = 2). The proportion of patients who received immunotherapy prior to sample collection was significantly higher for those with low-positive results compared to clear-positive results (88% vs. 50%, *p* = 0.0007) (Table 2). The median time from symptom onset of most recent attack to sample collection was significantly longer for those with low-positive results compared to clear-positive results (849 days vs. 17 days, *p* < 0.00001) (Table 2).

**Table 2:** Positive predictive value and sample collection timing of low-positive versus clear-positive anti-MOG results reported out from London Health Sciences Centre Clinical Immunology laboratory.

	All positive anti-MOG results *N* = 85	Low-positive anti-MOG results (Titer < 1:100) *N* = 47	Clear-positive anti-MOG results (Titer ≥ 100) *N* = 38	*p*-value*
PPV (overall)	82%	72%	95%	**0.009**
PPV (pediatric)	91%	89%	92%	>0.99
PPV (adult)	68%	47%	100%	**0.004**
Proportion of TP who received immunotherapy prior to sample collection	69%	88%	50%	**0.0007**
Median time from symptom onset to TP sample collection, days (range)	101.5 (1–2,793)	849 (7–2,793)	17 (1–2,405)	**<0.00001**

*p-value refers to comparison of difference between low-positive and clear-positive anti-MOG results.

MOG, myelin oligodendrocyte glycoprotein; PPV, positive predictive value; TP, true-positive.

Bolded values represent statistically significant results.

## Discussion

We found the PPV of positive serum fixed CBA anti-MOG results reported out by LHSC Clinical Immunology laboratory to be reasonably high at 82%. When stratified by low-positive versus clear-positive results as outlined by the 2023 MOGAD criteria, the PPV of low-positive anti-MOG results was significantly lower than that of clear-positive anti-MOG results. This finding is in line with studies using live CBA, which have found the PPV of low-positive anti-MOG results to be as low as 51% (8, 9). It also underscores the importance of critically examining low-positive fixed CBA anti-MOG results in patients with atypical disease phenotypes, and supports our current clinical laboratory practice of reporting out titers to help clinicians distinguish between low-positive and clear-positive anti-MOG results.

Notably, however, the difference in PPV between low-positive and clear-positive anti-MOG results was significant among adults tested in our laboratory, but not children. This discrepancy could indicate that the absolute difference in specificity between low-positive and clear-positive fixed CBA anti-MOG results reported out from our laboratory is relatively small, but translates to substantial differences in PPV when testing is performed in populations with a lower prevalence of MOGAD (e.g., adults with suspected inflammatory demyelinating disease, who likely have a lower prevalence of MOGAD than children with suspected inflammatory demyelinating disease) (14, 16–18). The potentially marginal difference in specificity between low-positive and clear-positive fixed CBA anti-MOG results may be a worthwhile focus of other laboratories who implement this test offering and opt to perform QA assessments of its utility in their own particular setting, especially those in resource-limited areas (25). Among patients with anti-MOG results that were classified as TP, the proportion who received immunotherapy prior to sample collection was significantly higher and median time from symptom onset to sample collection was significantly longer for those with low-positive compared to clear-positive results. This highlights that timing of sample collection relative to administration of immunotherapy as well as symptom onset may have a substantial impact on fixed CBA anti-MOG titers reported out from our laboratory, and indicates that testing should ideally be performed close to time of attack on samples collected prior to immunotherapy (19, 26).

There are several limitations to our assessment. Classification was based on retrospective review of electronic medical records, which were not necessarily uniform in their documentation. However, patient assessment by MS/Neuroimmunology specialists in the large majority of cases aided in ensuring completeness of documentation pertinent to MOGAD evaluation. We did not calculate PPV stratified by ordering provider (e.g., MS/Neuroimmunology specialist versus non-MS/Neuroimmunology specialist), because specialist input that may have guided the initial decision to order testing (e.g., via trainee supervision or consultant correspondence) could not be ascertained. As a QA assessment of fixed CBA anti-MOG titers being performed at LHSC Clinical Immunology laboratory, focus was placed on the diagnostic performance and interpretation of this recent test offering at our institution. This meant that more in-depth clinical characterization of patients with positive anti-MOG results, including analyses of disease phenotypes, monophasic versus relapsing disease and response to immunotherapy, was outside the scope of this assessment. Furthermore, assessment of PPV stratified by titers between 1:10 and 1:100 (e.g., 1:20, 1:40, 1:80) was not possible, as such titers are not performed at our institution. We also did not attempt to validate the 2023 MOGAD criteria, which was similarly outside the scope of this QA assessment but has been the focus of recent work by others (27).

We herein demonstrate diagnostic utility of anti-MOG titers by fixed CBA that are performed in our laboratory to distinguish between low-positive and clear-positive antibody results, indicating that our decision to offer this service in line with the 2023 MOGAD criteria represents an improvement in the quality of testing we provide to clinicians. We found that considering patient characteristics that inform likelihood of MOGAD as well as timing of sample collection is valuable to the interpretation of anti-MOG fixed CBA titers reported out from our laboratory, which we plan to comment upon in our reports to clinicians moving forward. Other clinical laboratories that are considering offering serum anti-MOG titers by fixed CBA, which may differ from our laboratory in their qualitative reporting of immunofluorescence intensity as well as the region they service, would benefit from performing similar assessments dedicated to assuring the quality of this offering in their own institutional setting.

## Data availability statement

The original contributions presented in the study are included in the article/supplementary material, further inquiries can be directed to the corresponding author.

## Ethics statement

Ethical approval was not required for the study involving humans in accordance with the local legislation and institutional requirements. Written informed consent to participate in this study was not required from the participants or the participants’ legal guardians/next of kin in accordance with the national legislation and the institutional requirements.

## Author contributions

AB: Conceptualization, Formal analysis, Investigation, Methodology, Writing – original draft. DLR: Conceptualization, Writing – review & editing. LY: Conceptualization, Writing – review & editing. EY: Conceptualization, Writing – review & editing.
